# Retraction: Mechanisms for *Alternaria alternata* function in the skin during induction of peanut allergy in neonatal mice with skin barrier mutations

**DOI:** 10.3389/falgy.2026.1842963

**Published:** 2026-04-02

**Authors:** Frontiers Editorial Office

The journal retracts the 06 September 2021 article cited above.

Following publication, concerns were raised by Indiana University regarding the integrity of data presented in several figures. An institutional investigation found evidence of data falsification in the preparation of Figures 4A, 4D, 4E, 6B and 6C, including the omission of data points and the reporting of results below the standard optical density threshold of the ELISA assay. The investigation concluded that the underlying data were not reliable and recommended retraction of the article to correct the scientific record.

This retraction was approved by the Chief Editors of Frontiers in Allergy and the Chief Executive Editor of Frontiers. The authors have not responded to correspondence regarding this retraction.

